# Prevalence, Preventive Methods, Health-related Outcomes, and Correlates of Sexual Behaviours among Adolescents in Malinyi District Council, Morogoro region, Tanzania

**DOI:** 10.24248/eahrj.v9i1.828

**Published:** 2025-09-30

**Authors:** Magdalena G. Dinawi, Walter C. Millanzi, Patricia Z. Herman

**Affiliations:** a Ministry of health and social affairs, Department of DPS & HPS; b Department of Nursing Management and Education, University of Dodoma; c Institute of Health and Allied Sciences, Ruaha Catholic University.

## Abstract

**Background:**

Social media, peers, teachers, parents, religious leaders, and close family members are just a few of the many sources of sexual and reproductive health (SRH) information, education, related healthcare services, and rights that teens are inundated with for reproductive health, including safe sexual behaviors. This study evaluated the prevalence, preventive methods, health-related outcomes, and correlates of sexual behaviors among adolescents in the Malinyi district council, Morogoro region, Tanzania.

**Methods:**

An analytical cross-sectional study was conducted on 331 randomly selected in-school adolescents. The major data-gathering tool was interviewer-administered structured questions from prior studies. The data were analyzed using version 25 of the IBM Statistical Package for Social Sciences (SPSS), with a 95% confidence interval and a 5% significance level.

**Results:**

The average age of respondents was 13 years ± 0.91, with 54.1% being female. 86.0% of respondents were sexually active before the age of 19 years, of which 48.0% of them had sex with friends, 11.1% with mobile phone salespeople, 9.1% with motorcycle drivers, and 1.7% with their biological parents. 44.0% of adolescents reported using no any type of contraception during sexual activity, while 8.7% and 13.3% reported using plastic bags and Vaseline jelly, respectively. Adolescents who experienced verbal sexual jokes (AOR = 2.009; *p*<.05; 95%CI: 1.1.012–4.912), physical/tactile sexual jokes (AOR = 1.905, *p*<.05; 95%CI: 1.011–3.397), owning smartphones (AOR = 1.310; *p*<.05; 95%CI: 1.022–3.365), discussing family planning with friends (AOR = 2.300; *p*<.05; 95%CI: 1.013–3.321), and using social media platforms (AOR = 1.708; *p*<.05; 95%CI: 1.030–3.431) were at significant risk of early onset of unsafe sexual behaviors than others (*p*<.05).

**Conclusion:**

Early onset of sexual behaviors among adolescents is still prevalent. Engaging in sexual activities with individuals closest to them, as well as others who were their blood family, utilising medically unapproved means such as plastic bags and Vaseline jelly during sexual activities, are common among adolescents. The study suggests system-wide school-based interventions to provide adolescents with age-appropriate and comprehensive SRH education and information for the delayed onset of sexual activities.

## BACKGROUND

The foundation of adolescents’ health is to enhance access to and availability of comprehensive sexual and reproductive health (SRH) education for relevant and timely knowledge and soft skills about SRH and its services.^1^ Adolescents between the ages of 10 and 19 years have proportionally equal rights to education and information regarding pubertal changes, the right time to begin and engage in safe sexual relationships, and what, when, and from whom to access SRH services, regardless of geographical locations and ethnicities.^2^ However, they are often overloaded with sexual and reproductive health (SRH) information, education, and related healthcare services from a variety of sources, including social media, peers, teachers, parents, religious leaders, and/or close relatives, all of which are deemed untrustworthy, insufficiently regulated, and inadequately monitored.^3,4^

Some scholars argue that adolescents living in rural areas are more susceptible to missed opportunities for age-appropriate SRH education, information, and related health services that are closely linked with their exposure to sexual masculinities, sexual violence, and early onset of unsafe sexual behaviors that put them at risk for teenage pregnancies, STIs like HIV, and school dropouts.^5,6^ Compulsive sexual behaviors in adolescents are primarily argued to drive them to the behaviors because they are psychological immaturity, which makes it difficult for them to choose to postpone having sex, remain faithful, or say “No” to sexual activities and thus, act carelessly and erratically.^7^ Nevertheless, adolescents may sometimes engage in multiple sexual partners, sex as a source of income, unprotected sexual contact with peers or adults, and/or abortions, though the extent of these behaviors and their effects on health outcomes may be unclear.^8^

Scholars have found that, despite the continuum of guidance and support provided by national health policies, health strategic plans, parents, immediate family members, teachers at schools, administrative bodies in camps, and/or religious leaders, the majority of adolescents are now beginning sexual relationships as early as nine years old.^9,10^ Up to 60% of adolescents worldwide and 69% of those in Sub-Saharan African countries engage in risky sexual behavior as a result of their inability to make mature and educated judgments about sexual behaviors^[11,12]^ some engage in risky sexual behaviours. Such risky behaviours expose adolescents to unintended pregnancy and sexually transmitted infections (STIs. Moreover, it is estimated that of the 1.7 million new HIV infections in 2019, or nearly 5,000 new HIV infections per day, 1.5 million are adults, while adolescents account for 150,000. Sub-Saharan Africa hosted approximately 182,599 (73%) of the 250,000 young people of school age who had recently contracted HIV.^13–15^

Tanzania, like other developing nations, has 4.8% of the world's HIV-positive population, with a significant rise in prevalence from 1.3 million in 2010 to 1.7 million in 2019.^16,17^ Despite Tanzania's HIV prevalence among those aged 15 to 49 declining from 5.1% in 2014 to 4.8% in 2019, 5.8% (N = 104,400) of the world's adolescents with HIV (N = 1,800,000) are found there.^18^ According to estimates, 10,000 of the 77,000 new HIV infections in the nation each year are in adolescents aged 10 to 19 years. Approximately 8,600 new HIV infections occur between the ages of zero and middle adolescence (0 to 14 years), and 93,000 children in the same age group are HIV-positive of which of the 99,000 adolescents with HIV between the ages of 10 and 19, approximately 57,000 are teenage girls while out of the 27,000 expected fatalities in the country, 5,900 children die as a result of AIDS-related causes.^19,20^

Despite some progress in lowering the rate of new STIs and HIV among adolescents, the reported trend may indicate that the current strategies are either not sustainable, are not reaching a sizable enough group of adolescents, or something is missing from the planning, execution, or evaluation of these strategies.^21,22^ In addition to the increase in STIs and HIV among adolescents, unintended pregnancy among adolescents is a problem.^23,24^ According to the World Health Organization (WHO), more than 2.5 million (12%) females become mothers by the age of 16, and approximately 21 million girls (15–19 years old) become pregnant each year, giving birth to 12 million children whereas East Africa appears to have a high frequency (21.5%), while Northern Africa has a low incidence (9.2%).^25^ Tanzania (22.8%) and Uganda (23.8%) have the highest rates of adolescent pregnancies, according to reports from East African countries, while Rwanda (7.3%) and Ethiopia (12.4%) have the lowest rates when compared to other African countries.^14^

Using inclusive, holistic strategies that prioritize young people in the fight against the early onset of unsafe sexual behaviors at home, school, camp, and religious institutions can help win a significant battle against the early onset of sexual intercourse, early marriages, and sexual violence.^6,26^ Based on the available research, it is unclear whether the aforementioned organizations provide adolescents with complete, timely, age-appropriate, and friendly SRH information/education in addition to their services for safe sexual practices.^27,28^ There seem to be several unanswered questions, such as what is the prevalence of sexual behaviors, and how early and with whom do adolescents in Malinyi District Council initiate sex? Which preventive methods do adolescents at Malinyi District Council use for safe sex? Which adverse health-related outcomes do adolescents in Malinyi District Council face? What are the drivers of early sexual behaviors among adolescents in Malinyi District Council? Thus, this study aimed to evaluate the prevalence, preventive methods, health-related outcomes, and correlates of sexual behaviors among adolescents in the Malinyi district council, Morogoro region, Tanzania.

## METHODS

This study was conducted using the guidelines and standards for undergraduate and graduate programs at the institution, which serve as a foundation for research while adhering to ethical considerations to meet national and international research ethics.^29^ This study did not include experiments on higher vertebrates or living vertebrates as part of an intervention. The Institutional Project Review and Approval Committee (IPRAC) of Malinyi District Council approved the study with a letter of approval, reference MDC/ADM/H.10/33, because it complied with the institution's regulations from 28^th^ to 29^th^ September 2020, and the study was conducted from 30^th^ to 02^nd^ October 2020. This work did not receive any specific grant from funding agencies in the public, commercial, or not-for-profit sectors. Authors had access to the information that could identify individual respondents during or after data collection.

### Study Area

The study was conducted in Tanzania's Morogoro Region's Malinyi District Council, which is the southernmost of the nine (9) councils, because according to sexuality data from the district, the majority of adolescents engaged in sexual activity during their early years of age. In 2020, the district had 51.4% more adolescent pregnancies than other districts in the region, and the regional rate of male sexual assault was 9.7%.

### Study Design

A school-based analytical cross-sectional study using a quantitative research approach was conducted to collect data at a single point in time and determine the association between study variables among adolescents in the Malinyi District Council, Morogoro region, Tanzania.

### Sample Size Estimation

Considering the recommendations of previous researchers, to establish the minimum sample size for the study at a 95% confidence level, the following approaches were used. In the study by Millanzi et al..^27^, the prevalence used to calculate the sample size was 26.7% of the respondents, as was found in their study, who reported they had at least one conversation with their biological parents regarding sexual and reproductive health during their lives.



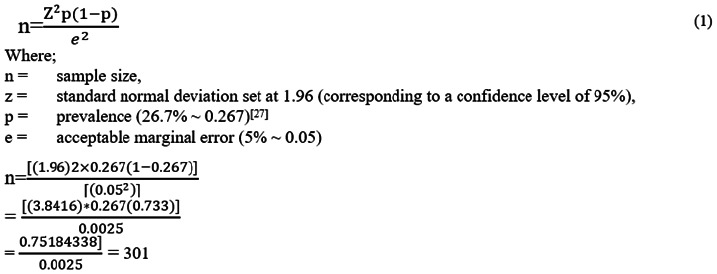



The non-response rate was set at 10% of the determined sample size, equivalent to 30 respondents. Thus, the minimum sample size of this study was n = 331 respondents. As recommended by previous scholars, the sample was proportionally distributed in strata based on the selected secondary schools, classes, and year of study using the proportionate formula (ni = Pi × n/P).

### Eligibility Criteria and Informed Consent

As benchmarked from some previous studies^30–37^, Participation in this study was voluntary, and adolescents aged between 10 and 19 years in secondary schools (who were admitted, registered, and lived on or off campus) were recruited. The lead investigator approached legally authorized relatives, instructors, or parents who consented on behalf of all minors under 18 years old. It was requested that school administrators write letters informing legally acceptable parents and/or relatives about the study and schedule meetings with the research team so that they could voluntarily offer written informed permission on behalf of their children after being briefed on the study's details.

### Sampling Technique

Ten (10) secondary schools were selected through a simple random selection procedure and a lottery system as suggested by some previous scholars.^38–40^ A stratified random sampling procedure divided them into government-owned (n = 5) and privately owned (n = 5) schools. In addition, classes were selected using a stratified sampling strategy^41^, and a random numbers table sampling method^42^ was used to obtain a minimum sample of study respondents. To achieve the required minimum sample size for this study, all study respondents were listed on paper and assigned numbers ranging from one to the proportionate number in that specific school. The researcher then closed their eyes, pointed to a random point on the number chart, and moved in any direction until they found the number on the list. If a respondent's number appeared on the list, it was saved; otherwise, it was discarded. This procedure was repeated until there was an adequate sample of usable numbers with no repetitions.

### Data Collection Procedures

The principal investigator used interviewer-administered structured questionnaires from previous studies^[43,44]^ Development and Research Training in Human Reproduction (HRP to collect data on the prevalence and underlying factors influencing early sexual behavior initiation, and adolescent use of sexual and reproductive health services in Tanzania's Malinyi District Council. The day of the data collection meeting was decided and scheduled after the sampling procedures, which were carried out at a separate, unoccupied location provided by the respective institutions to ensure privacy. Respondents sat in separate chairs to avoid responses sharing, copying, and pasting. Before beginning the questionnaire filling process, respondents were given brief instructions, and research team was on hand to supervise, answer questions, collect completed surveys, and secure them. To maintain confidentiality, respondents’ names were replaced with codes in the questionnaires. The questionnaires were completed in between thirty and forty-five minutes each.

### Data Collection Tools and Variable Measurements

The HIV/AIDS questionnaires and the World Health Organization's (WHO) illustrative Questionnaires for Interview Surveys with Young People served as the model for the data collection method employed in this study.^27,44^ Development and Research Training in Human Reproduction (HRP The questionnaires were divided into four sections, with component “A” asking about the sociodemographic characteristics profiles of the study respondents (n = 21 items). Part ‘B’ contained questions on sexual behavior alongside the onset of it (n = 15 items), such as *“Other adolescents tend to have sexual intercourse at least once in their life, what about you? Other adolescents tend to start sexual activities at an early age. What about you? Other adolescents tend to have sexual intercourse with boyfriends, with whom did you have sexual intercourse?”* just to mention a few.

Part ‘C’ asked about the methods they used to engage in safe sex (n = 5 items), such as “*Other adolescents tend to use family planning methods during the sexual act, what about you? Other adolescents tend to use condoms during the sexual act. Which protection method do you use?”* Part ‘D’ asked about whether respondents ever experienced any health-related outcomes from sexual activity at least in the past year before this study (n = 5 items), such as *“Other adolescents tend to experience health-related problems after the sexual act. Has this happened to you? Other adolescents tend to experience pain after the sexual act. What happened to you?”*

Items were structured with “Yes”/”No” responses. A one (1) value was assigned to a “Yes” response if the study respondent experienced the event assessed in that item; otherwise, a zero (0) value was assigned to a “No” response if they did not. Study respondents with high scores were regarded as having the behavior; they used the preventive method and experienced the health-related outcomes, otherwise not.

### Control of Bias

As recommended by some previous studies^45–48^, the respondents were selected using random sampling techniques and procedures to ensure that the information on the study topic was random. Nevertheless, to reduce halo bias, the study employed research assistants who were not from the sampled setting, while the study's obedience effect was addressed by putting researchers, data analysts, and respondents into separate independent groups. The study posed questions about sexual behaviors that occurred at least one year prior to the commencement of this study, assuming that adolescents could accurately recall relevant sexual events to control for recall bias. Additionally, the data collection tools were sourced from reliable origins and were standardized to inquire about adolescents’ sexual and reproductive health issues related to their real-life experiences, aiming to minimize information bias.

**Validity:** In this study, content validity was selected and ensured by developing relevant and effective research instruments. These instruments were then shared with statisticians and expert colleagues for feedback on their content suitability, sentence structure, language, and organization. The feedback indicated that the research tools needed to be translated into Swahili to align with the literacy levels of the study respondents and enhance the clarity, understanding, accuracy, and completeness of the information. Other factors, including content appropriateness, sentence structure, and item organization, remained unchanged. The tools were returned for final review, and no further revisions were necessary.

**Reliability:** To prevent information tampering, the primary investigator tested the research instruments on a 10% (n = 30) sample of the estimated sample size in a location other than the sampled study settings. Indicators include the items’ applicability, language appropriateness, clarity, and time required to complete the surveys. Ten contacted independent reviewers/observers were asked to rate the items’ relevance using the inter-observer rating method. A pre-test observation revealed that all topics were relevant, scoring between 9 and 10, while the language was acceptable and understandable, and the surveys would be completed in 30 to 60 minutes. To assess the tool's reliability, the pre-test results were subjected to a scale analysis, which revealed that the items assessing sexual behavior had a Cronbach's = 0.77. As recommended by previous researchers^[49,50]^, the tools were then considered reliable for the actual data collection.

### Data Analysis

Data were cleaned and descriptively analyzed using the IBM Statistical Package for Social Sciences (SPSS) computer software version 25. The sociodemographic profiles of the study respondents’ sexual behavior prevalence, abortion rates and techniques, and use of sexual and reproductive health services were quantitatively assessed and reported in frequencies and percentages. Binary and multinomial logistic regression models were used to determine the relationship between predictor variables and the outcomes of interest under study, with a 95% confidence interval and 5% significance level. There was no missing information/data in this study. This logistic regression model was employed for data analysis.



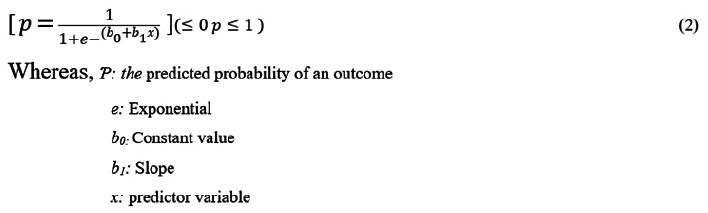



## RESULTS

**FIGURE 1:**
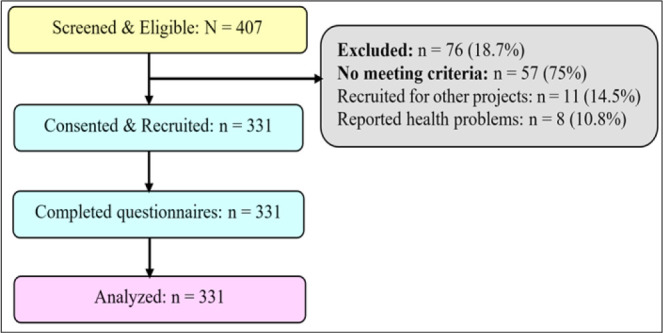
A Sample Size Flow Pattern of the Study Respondents (n=331)

As shown in Figure 1, given the minimum sample size and inclusion criteria, 81.3% (n = 331) agreed to par-ticipate. 18.7% (n = 76) of adolescents were excluded; 75% (n = 57) did not meet the inclusion criteria; 14.5% (n = 11) were recruited for other projects; and 10.5% (n = 8) reported health issues that would make providing information difficult. Because of the exclusion procedure being carried out before the start of data collection, only 331 study respondents finished the study with a 100% response rate, and their data were evaluated as a result.

### Socio-demographic Characteristics Profiles of the Study Respondents

Table 1 shows that 58.3% (n = 193) of respondents attended government-owned schools, with a mean age of 13±0.917 years. 41.4% (n = 137) and 30.5% (n = 101) of respondents were aged between 17 and 19 years and 13 and 16 years, respectively. It was discovered that 64.1% (n = 212) were living on campus, with 54.1% (n = 179) being females and 45.9% being males. Although 86.7% (n = 287) of the study respondents came from nuclear families, some (2.1%) and 8.8% (n = 29) lived with friends and relatives rather than their biological parents. Physical disabilities were reported by 19.0% (n = 63) of study respondents, whereas 19.0% (n = 63) and 10.6% (n = 35) of study respondents commuted to school on foot and by motorcycle, respectively.

**TABLE 1:** Distribution of Depression Related Aspects on the PHQ - 9 Scale

Variable	n(%)
School	
Government-owned	193 (58.3)
Private-owned	138 (41.7)
Age: Mean = 13±0.917 years	
10–12 years	93 (28.1)
13–16 years	101 (30.5)
17–19 years	137 (41.4)
Sex	
Male	152 (45.9)
Female	179 (54.1)
Physical/psychological disability	
Yes	63 (19.0)
No	268 (81.0)
Accommodation	
In-campus	212 (64.1)
Off-campus	119 (35.9)
Type of family at home	
Nuclear	287 (86.7)
Extended	44 (13.3)
Living with whom at home	
Both biological parents	159 (48.0)
Mother only	99 (30.0)
Father only	37 (11.1)
Relatives	29 (8.8)
Friends	7 (2.1)
Means of transport to school On foot	
Bicycle	63 (19.0)
Motorcycle	35 (10.6)
Public bus	12 (3.6)
Private car	9 (2.7)
Living in hostels/camps	212 (64.1)
Class	
1st year	98 (29.6)
2nd year	122 (36.9)
3rd year	111 (33.5)
Mobile phone ownership	
Yes	75 (22.7)
No	256 (77.3)
Type of mobile phone	
Cell phone	7 (2.1)
Smartphone	68 (20.6)
Not owning a mobile phone	256 (77.3)
Use of mobile phone	
Communication	43 (13.0)
Searching learning materials	3 (0.9)
Watching posted videos/photos	22 (6.7)
Playing games	7 (2.1)
Not owning a mobile phone	256 (77.3)
Social media platforms	
Facebook	9 (2.7)
Twitter	3 (0.9)
WhatsApp	42 (12.7)
Snap chat	12 (3.6)
Instagram	2 (0.6)
Telegram	0 (0.0)
Not in the social platform	263 (79.5)
Use of social media platforms	
Reading news	4 (0.9)
Chatting	47 (14.2)
Watching posted videos/photos	16 (4.8)
Sharing updates	1 (0.3)
Buying/selling products	0 (0.0)
Not in the social platform	263 (79.5)
Attended social ceremonies at night during the last study leave	
Traditional ceremonies/rituals	97 (29.3)
Birthdays	117 (35.4)
Wedding	51 (15.4)
Night music clubs/concerts	15 (4.5)
Entertainment shows	9 (2.7)
Never attended	42 (12.7)
Watching television	
Yes	324 (97.9)
No	7 (2.1)
Sessions watched televisions	
Movies	67 (20.2)
Dramas	116 (35.1)
News	21 (6.3)
Entertainments show	18 (5.5)
Music	65 (19.6)
Ubongo Kids/Cartoons	14 (4.2)
Playing games	23 (7.0)
Never watched television	7 (2.1)
Engaged in gambling	
Yes	124 (37.5)
No	207 (62.5)
Communicated with whom SRH matters	
Both parents	29 (8.8)
Father only	17 (5.0)
Mother only	99 (30.0)
Relatives	39 (11.8)
Teachers	8 (2.4)
Friends	48 (14.5)
Never communicated	91 (27.5)
Preferred individual to communicate SRH matters	
Both biological parents	9 (2.7)
Mother only	109 (32.9)
Father only	7 (2.1)
Relatives	19 (5.8)
Teachers	8 (2.4)
Friends	88 (26.6)
Never communicated	91 (27.5)
Topic communicated	
Puberty	15 (4.6)
STIs/HIV	97 (29.3)
Teenage pregnancies	26 (7.9)
Abortion	13 (3.9)
Fistula	3 (0.9)
Family planning methods	8 (2.4)
Cervical cancers	5 (1.5)
School dropouts from teenage pregnancies	61 (18.4)
Sexual relationships	12 (3.6)
Never communicated	91 (27.5)
Exposure to verbal sexual jokes	
Yes	137 (41.4)
No	194 (58.6)
Exposure to physical/tactile sexual jokes	
Yes	111 (33.5)
No	220 (66.5)
Exposure to training, seminars, workshops, or sessions about SRH matters	
Yes	106 (32.0)
No	225 (68.0)

Key: n: Frequency/count, %: Percentage, ≥50%: High proportion

Despite their small number, 22.7% (n = 75) of the study respondents owned mobile phones, with 20.6% (n = 68) owning smartphones, 13.0% (n = 43), 6.7% (n = 22), and 2.1% (n = 7) of them used their mobile phones for communication, watching videos/photos, and playing games. Study respondents who joined social media platforms such as Facebook, WhatsApp, Instagram, Snapchat, and/or Twitter, to name a few, accounted for 20.5% (n = 68), with 14.2% (n = 47) using the platforms for chatting and 4.8% (n = 16) for watching posted videos/photos. Furthermore, the table shows that 87.3% (n = 289) of the study respondents attended one or more ceremonies during their most recent study leave, while 35.4% (n = 117) attended birthdays, 29.3% (n = 97) attended traditional ceremonies/rituals, 4.5% (n = 15) attended night music clubs/concerts, and 15.4% (n = 51) attended weddings.

According to this study, 72.5% (n = 240) of respondents have discussed sexual and reproductive health issues with their biological parents, father only, mother only, relatives, teachers, and/or friends. Only 27.5% (n = 91) had never spoken with anyone about sexual and reproductive health. STIs (29.3%), school dropouts due to teenage pregnancies (18.4%), and teenage pregnancies (7.9%) were the most commonly communicated issues. However, the majority of study respondents preferred to discuss sexual and reproductive health issues with their mothers (32.9%) and friends (26.6%) rather than their fathers or other relatives whereas 41.4% (n = 137) and 33.5% (n = 111) of the study respondents had at least once encountered verbal and physical/tactile sexual jokes. Nonetheless, 32.0% (n = 106) of the study respondents had received some sexual and reproductive health training prior to this study, compared to 68.0% (n = 228) who had not.

### The prevalence and onset of sexual behaviors among the study respondents in the Malinyi district council

**FIGURE 2:**
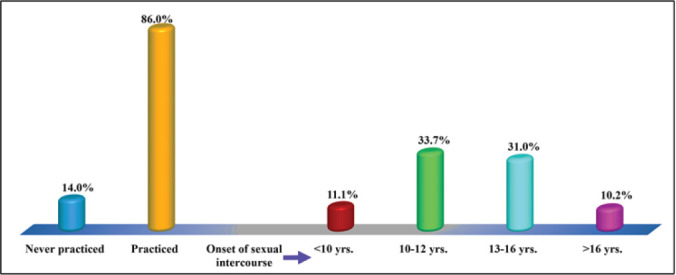
Prevalence and Onset of Sexual Behaviours among the Study Respondents in Malinyi District Council (n=331)

Figure 2 depicts the descriptive findings for the prevalence and timing of sexual intercourse among study respondents. The findings revealed that 86.0% (n = 285) of the study respondents were sexually active and engaged in sexual intercourse at least once during their adolescence stage, while 24.0% (n = 46) did not. Furthermore, study findings revealed that 33.7% (n = 96) of the study respondents initiated sexual intercourse at the age between 10 and 12 years, 31.0% (n = 88) at the age between 13 and 16 years, and some (11.1%) and (10.2%) at the age <10 years and >16 years, respectively.

### Proportional Distributions of Individuals with whom the Study Respondents had Sexual Intercourse in Malinyi District Council

**FIGURE 3:**
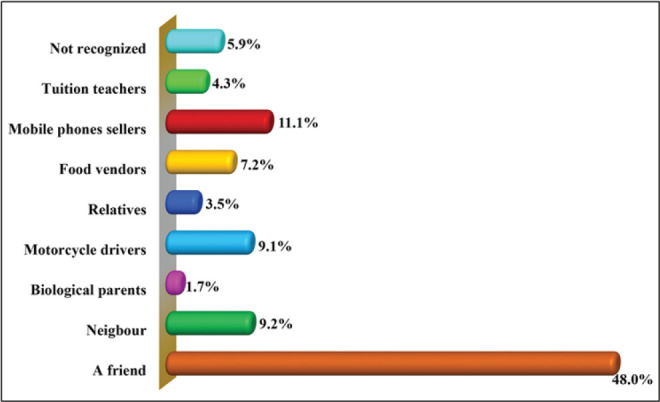
Proportional Distribution of Individuals whom the study Respondents in the Malinyi District Council had Sexual Intercourse Within Malinyi District Council (n=285)

Figure 3 shows that 5.3% (n = 15) of study respondents engaged in sexual intercourse with blood and close relatives, compared to 94.7% (n = 270) who did so with strangers including friends; mobile phone sellers; neighbors; motocycle riders and/or food vendors. Findings show that 48.0% (n = 137) of the study respondents had sexual encounters with friends, 11.1% (n = 37) with mobile phone sellers, 9.2% (n = 26) with neighbors, 9.1% (n = 25) with motorcycle drivers, and 7.2% (n = 20) with food vendors working in cafeterias, restaurants, and/or chip corners. Although they were few, 3.5% (n = 10) and 1.7% (n = 5) had sexual relationships with blood relatives and biological parents, respectively.

### Proportional distributions of different items/methods used by the study respondents to practice safe sexual intercourse in Malinyi District Council

**FIGURE 4:**
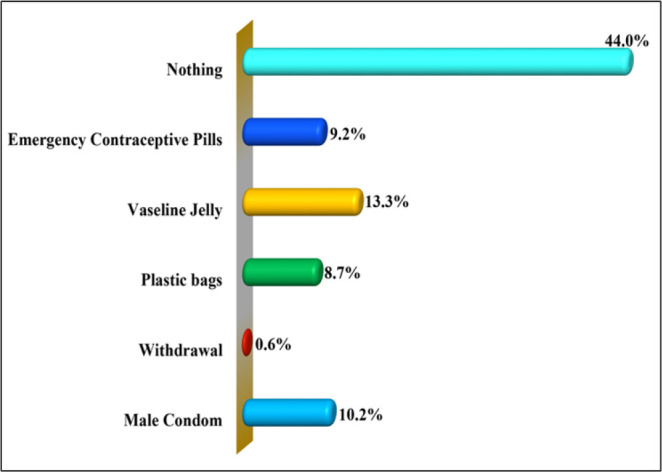
Proportional Distribution of Different Items Used by the Study Respondents to Practice Safe Sexual Intercourses (n=285)

**FIGURE 5:**
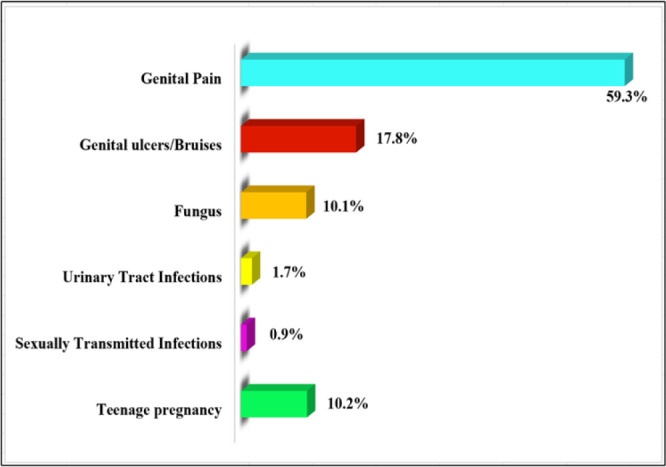
Proportional Distribution of the Outcomes Related to Unsafe Sexual Behaviours among In-School Adolescents in Malinyi District Council (n=285)

This study evaluated how study respondents engaged in safe sexual intercourse of which Figure 4 shows that 44.0% (n = 125) of them did not use any methods for safe sexual intercourse compared to 13.3% (n = 38) respondents who used Vaseline jelly, 10.2% (n = 29) male condoms, 9.2% (n = 26) emergency contraceptive pills, 8.7% (n = 25) plastic bags, and 0.6% (n = 2) withdrawal methods with their sexual partners. (Figure 5)

### Proportional distributions of the health-related outcomes to unsafe sexual behaviors among in-school adolescents in Malinyi District Council

Findings in Figure 5 indicate that out of 285 respondents who practiced sexual acts, 59.3% (n = 169) of them experienced genital pain during and after sexual intercourse, which is in contrast to 17.8% (n = 51) of them who experienced genital ulcers/bruises. Moreover, 10.2% (n = 29) and 10.1% (n = 28) of the respondents reported having had teenage pregnancy and fungus in comparison to 1.7% (n = 5) and 0.9% (n = 3) of them reported having experienced urinary tract infections (UTIs) and sexually transmitted infections (STIs) after sexual intercourse, respectively. (Figure 4)

### Factors associated with the onset of sexual behaviors among adolescents in Malinyi District Council

The study used a binary and multinomial logistic regression model to determine the relationship between variables. In this study, sociodemographic characteristics profiles of study respondents were treated as drivers that would demonstrate a significant effect of the dependent variable behaviors, in this case, the onset of sexual behaviours. Adolescents attending government-owned schools are one and a higher risk by one times more likely to develop sexual behaviors compared to those attending private schools (AOR = 1.015; *p*<.05; 95%CI: 1.008–3.964). Adolescents aged 10–12 years (AOR = 1.391; *p*<.05; 95%CI: 0.699, 2.675) and 13–16 years (AOR = 1.780; *p*<.05; 95%CI: 1.003–4.334) were one time more likely to engage in sexual behaviors than other age groups. Moreover, the study found that female respondents, those with physical disabilities, and those living off-campus were one times more likely to engage in sexual intercourse early in their lives than their counterparts (AOR = 1.451; *p*<.05; 95%CI: 1.002–3.462), (AOR = 1.611; *p*<.05; 95%CI: 1.052–3.871), and (AOR = 1.022; *p*<.05; 95%CI: 1.013–3.992), respectively. Nevertheless, living with a biological mother only (AOR = 1.072; *p*<.05; 95%CI: 1.143–2.079), biological father only (AOR = 1.033; *p*<.05; 95%CI: 1.103–2.446), and blood relatives (AOR = 1.034; *p*<.05; 95%CI: 1.003–2.798) were one time more likely to initiate sexual behaviors at early ages than their counterparts respectively. Additionally, adolescents who were living with friends (AOR = 2.433; *p*<.05; 95%CI: 1.173–6.446) were two times more likely to start sexual behaviors as compared to other adolescents.

On the other hand, this study found that walking on foot (AOR = 1.061; *p*<.05; 95%CI: 1.561–2.703) and using motorcycles (AOR = 1.098; *p*<.05; 95%CI: 1.183–2.807) were one time more likely to catalyze the onset of sexual behaviors among adolescents as compared to when they would use public transport, private car, and/or living on campus (*p*>.05). Adolescents who were in their second year of studies were one times more likely to engage in sexual intercourse (AOR = 1.665; *p*<.05; 95%CI: 1.015–3.220) than their counterparts in other years of studies. Mobile phone ownership and use were also evaluated for their influence on the onset of sexual intercourse among study respondents. The study found adolescents who owned mobile phones (AOR = 1.883; *p*<.05; 95%CI: 1.022–3.365) alongside the type of mobile phones (AOR = 1.310; *p*<.05; 95%CI: 1.050–2.944), and using them for watching video/photos (AOR = 1.121; *p*<.05; 95%CI: 1.033–2.698) were one time more likely to initiate sexual intercourse at early ages of their lives. 

Adolescents who watched posted videos/photos on social platforms were one time more likely to engage in influencing the onset of sexual intercourse (AOR = 1.708; *p*<.05; 95%CI: 1.033–2.698) than those who used them for other purposes, such as chatting. Nevertheless, adolescents who watched television (AOR = 1.039, *p*<.05; 95%CI: 1.003–2.655) and music sessions (AOR = 1.784, *p*<.05; 95%CI: 1.022–3.902) were one time more likely to initiate sexual intercourse during adolescence than those who did not. Findings in Table 2 shows that adolescents who were attending ceremonies such as traditional rituals (AOR = 1.032; *p*<.05; 95%CI: 1.004–2.671), birthdays (AOR = 1.307; *p*<.05; 95%CI: 1.143–2.109), weddings (AOR = 1.008; *p*<.05; 95%CI: 1.007–2.570), and night clubs/concerts (AOR = 1.799; *p*<.05; 95%CI: 1.042–3.021) were one times more likely to start sexual intercourse early in their lives than when not they would not attend them.

**TABLE 2:** Factors Associated with the Onset of Sexual Behaviours Among Adolescents in Malinyi District Council (n = 331)

Variable	COR	*p-value*	95%CI		AOR	*p-value*	95%	
			Low	Upper			Low	Upper
School								
Government-owned	1.767	.023	1.211	4.112	1.015	.031	1.008	3.964
Private-owned	1							
Age: Mean								
10–12 years	1.843	.019	1.734	3.111	1.391	.027	1.099	2.675
13–16 years	2.015	.007	1.032	5.217	1.780	.010	1.003	4.334
17–19 years	1							
Sex								
Female	2.034	.026	1.022	5.002	1.451	.038	1.002	3.462
Male	1							
Physical/psychological disability								
Yes	2.043	.002	1.120	5.101	1.611	.018	1.052	3.871
No	1							
Accommodation								
Off-campus	1.912	.028	1.390	4.232	1.022	.041	1.013	3.992
In-campus	1							
Type of family at home								
Nuclear	0.804	.105	0.065	1.773	0.591	.121	0.052	1.789
Extended	1							
Living with whom at home								
Friends	3.767	.005	1.385	8.545	2.433	.011	1.173	6.446
Mother only	1.541	.013	1.273	2.668	1.072	.020	1.143	2.079
Father only	1.767	.025	1.485	3.545	1.033	.031	1.103	2.446
Relatives	1.494	.017	1.159	3.991	1.034	.021	1.003	2.798
Both biological parents	1							
Means of transport to school								
On foot	1.404	.033	1.155	3.007	1.061	.044	1.561	2.703
Bicycle	0.876	.071	0.093	1.658	0.673	.088	0.078	1.574
Motorcycle	1.662	.012	1.481	2.990	1.098	.021	1.183	2.807
Public bus	0.810	.091	0.075	1.097	0.699	.112	0.057	1.081
Private car	0.337	.210	0.049	1.092	0.158	.262	0.032	1.057
Living in hostels/camps	1							
Class								
1st year	0.748	.104	0.226	1.903	0.575	.151	0.042	1.377
2nd year	2.587	.003	1.087	4.338	1.665	.015	1.015	3.220
3rd year	1							
Mobile phone ownership								
Yes	2.210	.007	1.210	4.094	1.883	.017	1.022	3.365
No	1							
Type of mobile phone								
Cell phone	0.783	.130	0.043	1.212	0.567	.146	0.019	1.030
Smartphone	1.764	.021	1.295	3.216	1.310	.033	1.050	2.944
Not owning a mobile phone	1							
Use of a mobile phone								
Communication	0.867	.304	0.322	1.523	0.784	.313	0.130	1.334
Searching learning materials	0.831	.300	0.207	1.420	0.583	.311	0.189	1.283
Watching videos/photos	1.774	.037	1.212	3.643	1.121	.046	1.033	2.698
Playing games	1							
Social platforms								
Facebook	1.904	.103	0.505	3.702	1.033	.125	0.201	2.307
Twitter	0.647	.141	0.024	1.840	0.467	.231	0.018	1.403
WhatsApp	1.222	.112	0.754	3.210	1.003	.129	0.383	2098
Snap chat	1.310	.201	0.834	2.744	1.004	.302	0.307	1.185
Instagram	2.777	.191	1.093	4.831	1.662	.205	0.032	1.505
Telegram	0.324	.071	0.076	1.885	0.089	.102	0.032	1.707
Not in the social platform	1							
Use of social media platforms								
Reading news	0.786	.133	0.232	1.326	0.669	.141	0.105	1.406
Chatting	0.883	.110	0.229	1.804	0.730	.125	0.106	1.451
Watching posted videos/photos	2.477	.007	1.302	5.323	1.708	.016	1.030	3.431
Sharing updates	1.747	.107	0.912	3.023	1.044	.114	0.701	2.904
Buying/selling products	1.578	.173	0.702	3.021	1.387	.187	0.533	2.053
Not in the social platform	1							
Attended social ceremonies during the last study leave								
Traditional ceremonies	1.113	.031	1.048	2.880	1.032	.042	1.004	2.671
Birthdays	1.533	.033	1.242	3.089	1.307	.040	1.143	2.109
Wedding	1.142	.025	1.029	2.775	1.008	.034	1.007	2.570
Night music clubs	2.210	.008	1.141	5.302	1.799	.017	1.042	3.021
Entertainment shows	0.974	.102	0.534	1.373	0.802	.120	0.304	1.104
Never attended	1							
Watching television								
Yes	1.142	.022	1.012	2.794	1.039	.034	1.003	2.655
No	1							
Sessions watched television								
Movies	0.664	.071	0.488	1.789	0.553	.084	0.207	1.547
Dramas	1.912	.103	0.803	3.021	1.655	.113	0.305	2.034
News	0.955	.254	0.771	2.894	0.805	.270	0.187	1.400
Entertainments show	1.842	.080	0.769	3.005	1.243	.101	0.574	2.103
Music	2.659	.037	1.055	5.202	1.784	.042	1.022	3.902
Ubongo kids/cartoons	1.902	.170	0.785	3.788	1.067	.182	0.538	2.200
Playing games	1.222	.109	0.786	3.201	1.037	.116	0.567	2.373
Never watched television	1							
Engaged in gambling								
Yes	0.881	.063	0.530	1.736	0.634	.132	0.375	1.603
No	1							
Communicated with whom SRH matters								
Both parents	1.042	.077	0.784	2.881	0.930	.082	0.693	1.992
Father only	1.843	.065	0.794	3.121	1.655	.090	0.652	2.849
Mother only	0.983	.069	0.621	1.783	0.828	.107	0.467	1.653
Relatives	1.731	.102	0.893	3.621	1.660	.113	0.731	3.002
Teachers	0.892	.079	0.384	1.833	0.703	.120	0.202	1.798
Friends	2.031	.020	1.053	4.210	1.902	.033	1.013	3.321
Never communicated 1								
Preferred individual to communicate SRH matters								
Both biological parents	1.663	.068	0.884	3.210	1.329	.073	0.644	2.642
Mother only	0.995	.084	0.803	1.856	0.808	.100	0.685	1.743
Father only	0.786	.075	0.404	1.774	0.684	.081	0.230	1.434
Relatives	1.110	.132	0.903	2.413	1.011	.142	0.744	1.941
Teachers	0.883	.093	0.742	1.794	0.689	.104	0.574	1.633
Friends	2.740	.021	1.103	5.921	1.740	.037	1.084	3.221
Never communicated	1							
Topic communicated								
Puberty	0.984	.072	0.871	1.864	0.823	.080	0.623	1.723
STIs/HIV	1.067	.183	0.783	2.654	0.783	.199	0.541	1.784
Teenage pregnancies	0.883	.082	0.711	1.889	0.749	.092	0.544	1.674
Abortion	0.744	.103	0.503	1.720	0.648	.119	0.357	1.554
Fistula	0.842	.112	0.753	1.993	0.650	.118	0.553	1.840
Family planning methods	2.932	.038	1.134	5.320	2.300	.044	1.081	4.202
Cervical cancers	1.332	.077	0.802	2.748	1.219	.083	0.622	1.894
School dropouts from teenage pregnancies	0.942	.123	0.730	1.839	0.647	.133	0.583	1.679
Sexual relationships 2.001		.019	1.682	4.830	1.783	.036	1.022	3.021
Never communicated	1							
Exposure to verbal sexual jokes								
Yes	3.110	.002	1.032	7.025	2.009	.018	1.012	4.912
No	1							
Exposure to physical/Tactile sexual jokes								
Yes	2.993	.008	1.184	5.339	1.905	.021	1.011	3.397
No	1							
Exposure to training, seminar, workshop, or session about SRH matters								
No	3.887	.001	1.403	7.893	2.733	.012	1.131	6.679
Yes	1							

Key

COR ≥1 and *p*<0.05: a positive predictor of the outcome variable when not controlled for other factors

AOR ≥1 and *p*<0.05: a positive predictor of the outcome variable when controlled with other factors (The final finding to be reported) p<0.05: Significant association between variables

Source: Field data (2023)

Furthermore, adolescents who had opportunities to communicate about sexual and reproductive health matters with friends were one times as likely to engage in sexual intercourse during adolescence (AOR = 1.902; *p*<.05; 95%CI: 1.013–3.321) compared to those who communicated SRH matters with their biological parents, relatives, or teachers. Adolescents who had opportunities to communicate sexual and reproductive health topics, such as family planning, with strangers or biological parents/caregivers (AOR = 2.300; *p*<.05; 95%CI: 1.081–4.202); adolescents who experienced verbal sexual jokes (AOR = 2.009; *p* <.05; 95%CI: 1.1.012–4.912) and those who had not previously received sexual and reproductive health education (AOR = 2.733; *p*<.05; 95%CI: 1.131–6.679) were two times more likely to engage in sexual intercourses early in their lives than other adolescents who were not. Moreover, findings demonstrated that adolescents who were in sexual relationships (AOR = 1.783; *p*<.05; 95%CI: 1.022–3.021), and those who experiences physical/tactile sexual jokes (AOR = 1.905; *p* <.05; 95%CI: 1.011–3.397) were one times more likely to initiate sexual intercourse at early ages of their lives against adolescents who had not.

## DISCUSSION

The study's findings revealed that the prevalence and early initiation of sexual activity among in-school adolescents were significantly higher. The majority of them were sexually active even below the age of ten years of whom they had sexual relationships with strangers, including friends, neighbors, motorcycle riders, and food vendors working in cafeterias, restaurants, and/or chips corners, while some of them did so with their biological parents and other blood relatives. The findings may convey the message that adolescents are not always to be blamed for the development of sexual behaviors; they are sometimes coerced by elderly people without their consent or protection and/or tempted by the foods or material possessions of those who hold or possess them. The trend would expose them to genital pain; genital ulcers/bruises; teenage pregnancies, fungus; STIs, and UTIs at much younger ages than most families expected or recommended by national health policy and health strategic plans.

While many adolescents did not engage in safe sexual activity with their partners, some did by using male condoms, emergency contraception, Vaseline jelly, plastic bags, or withdrawal methods. If the studied adolescents used nothing or an un-recommended/unauthorized method, such as plastic bags and Vaseline jelly, it could indicate that they were not adequately informed about sexual and reproductive health information, education, and related healthcare services to make informed, reasoned, and responsible decisions about sexual relationships and sexual intercourse. According to socio-demographic characteristics profile data, some adolescents continue to struggle with external factors such as being coerced or tempted by strangers (including friends, neighbors, motorcycle riders, and food vendors working in cafeterias, restaurants, and/or chips corners) to engage in sexual contact, relationships, and/or unprotected sexual activity while traveling to school, which contributes to the prevalence of early sexual behavior onset among them.

Nonetheless, adolescents with smartphones used them to communicate and view uploaded videos and images on online social platforms, implying that they had hidden opportunities to obtain mobile phones by themselves, or from strangers, possibly in exchange for sexual relations or labour activities, despite ongoing and close inspections and monitoring by school officials, parents, and/or close relatives. Both the in-school and out-of-school adolescents appeared to hold tendencies to hide their mobile phones in places where teachers in school or parents/caregivers at home could not discover them. They also appeared to have an unnoticed timing of using them. Based on this finding, the study contends that adolescents may have developed advanced cheating methods over parents, teachers, and other close family members at home, a behavioural trend that must be addressed; otherwise, the prevalence of early sexual behaviour onset among adolescents in school may persist.

Nevertheless, although the majority of adolescents had an open opportunity to discuss sexual and reproductive health issues with anyone or on social media platforms, most of them preferred to discuss sexual and reproductive health issues with their mothers and friends rather than their fathers or other close relatives. They preferred talks on topics such as STIs, teenage pregnancies, and/or adolescent pregnancy-related school dropouts. Moreover, some adolescents had access to sexual and reproductive health education from health facilities and television or radio, while others had never received sexual and reproductive health training or spoken with anyone about it. Despite being exposed to SRH training and/or communications, sexual behavior was nevertheless widely prevalent among them, which may probably imply that education and communication were insufficient to mold sexual behaviors, but rather, it appears that they encouraged them to try sexual Behaviours.

On the other hand, the findings of this study signpost that during their most recent study break from their respective schools to homes, the adolescents seemed to have opportunities to attend one or more public ceremonies, such as weddings, birthday celebrations, and traditional rituals. According to the reported behavioral situation, adolescents were occasionally released into a high-risk environment, causing them to be tempted to initiate sexual behaviors by either biological relatives or strangers against their will, under the influence of abused drugs, peer pressure, or coerced sexual encounters. The current study's findings are consistent with some scholarly studies, including those of Kushal *et al.*,^51^ in fifty countries, and Mesele *et al.*,^52^ in Ethiopia, who discovered that many adolescents begin having sexual relations before the age of ten (early adolescence stage), or before they reach the middle or late phases of adolescence, respectively.

Similarly, Waktole^53^ discovered in Ethiopia that adolescents began having sex before reaching middle adolescence due to their age, social media exposure, sociocultural norms, poverty, and pornographic viewing. The new study acknowledges and validates previous research findings that not all adolescents engage in sexual activity voluntarily; rather, some experience sexual masculinity, aggression, exploitation, victimization, and low socioeconomic backgrounds. As a result, not all initiations of sexual activity will be the result of coercion or temptation; some may occur voluntarily or on purpose, particularly in the middle and late stages of adolescence. Adolescents may have begun and continue to engage in sexual activity with one or more people/multiple sexual partners, including but not limited to biological parents/blood relatives, and strangers such as schoolteachers, peers, vendors, and/or shopkeepers on a voluntary or coerced basis.

In line with the findings of this study, Achen *et al.*,^54^ discovered in Uganda that many adolescents engage in early sexual activity as a result of adult sexual exploitation and masculinity. Most adolescents would engage in sexual activity with people older or younger than them to verbally respond to sexual jokes from friends or adults, or in exchange for monetary gain, because they are in the irresponsible stage and want to try the forbidden behavior. According to the findings of this study, adolescents who experience sexual assault, exploitation, or machismo may decide not to take any form of contraception to ensure they have safe sexual encounters. Given that most adolescents in developing countries, such as Tanzania, have low socioeconomic status, some may choose to practice safe sexual relations with materials that have not been scientifically proven to work, such as plastic bags or Vaseline jelly.

Variables such as the accommodation status of adolescents enrolled in school and the initiation of sexual behavior found in this study are consistent with other academic studies that discovered a statistically significant correlation between the variables under consideration. Tarkang *et al.*,^55^ and Watsi *et al.*,^56^ demonstrate that in-school adolescents who live off-campus face difficulties in avoiding sexual temptations and coercive relationships with peers and/or friends on the way to school in exchange for goods such as money, food, or transportation. However, previous studies’ findings are consistent with those of this study, as they show that adolescents who attend school off-campus are more likely to engage in sexual activity because they may feel unconstrained on their way home from school.

Close and continuous teacher supervision and control in schools, as well as a supportive parenting style, have been argued by scholars to be significant and protective variables for the early initiation of sexual behavior in adolescents. The findings of this study have demonstrated a link between the onset of sexual behaviors and adolescents’ proclivity to use mobile phones at home, in class, or on social media platforms where they are often tempted to watch sexually explicit dramas; films; stories; videos; cartoons; and/or pictures when using mobile electronic devices like laptops; computers; iPads; or smartphones in an unrestricted or unsupervised manner. Adolescents exposed to this type of mobile electronic device use sometimes find themselves doing or attempting to perform what they have seen in movies, videos, images, or sexually explicit stories, eventually becoming a habit because sexual and reproductive health issues from online sources are considered inappropriate for adolescents and should be handled accordingly.

Furthermore, according to social-cultural norms and household standards, attending ceremonies is more appropriate for adults than for children, including adolescents.^28,57^ However, findings of this study have indicated that adolescents are occasionally exposed to ceremonies such as birthdays, weddings, nightclubs, and/or traditional rites where they observe, learn, and risk imitating what adults do there, which can sometimes include sexual concerns or occurrences. Provided the loss of strong and sustainable cultural norms, beliefs, and dress codes that encourage adolescents to adopt and practice the same in private, may catalyze early initiation of sexual behaviors, among them. Adolescents’ ability to communicate about their sexual and reproductive health with those around them is critical to their development into healthy adults. Although parents, teachers, close relatives, and adolescents have embraced the current trend of SRH communications, its acceptance and implementation do not correspond to the prevalence of sexual behavior onset among adolescents.

Furthermore, scholars ^58–60^ have argued that adolescents who receive appropriate and comprehensive sexual and reproductive health information, education, and related healthcare services may gain the confidence to assertively say “No” to the onset of sexual relationships and sexual behavior at a young age and maintain that stance. In contrast to previous research, this study discovered that adolescents exhibited early-onset sexual behaviors despite exposure to SRH conversations with adults, social media, and peers. The situation may indicate the need for additional, innovative treatments based on research that prioritizes adolescent respondents’ sociodemographic characteristics.

## CONCLUSION

Early initiation of sexual behaviors among adolescents has been found in this study to be a problem of public concern, with some of them having sex with their biological parents, immediate family members, strangers, including peers, motorcycle drivers, food vendors, and/or smartphone salespeople. Extrinsic reasons, which sometimes spiral out of control such as the desire for mobile phones, joining and using social media platforms for communication; chatting; watching dramas and cartoons; posting pictures and videos, and/or playing games without the supervision of teachers, parents, or close relatives were among the drivers for unsafe sexual behaviors among adolescents. Most adolescents appeared to use medically unproven protective materials such as plastic bags and Vaseline jelly during sexual intercourse, which may imply that they are inadequately informed about the availability and accessibility of sexual and reproductive health information, education, and healthcare services in health facilities.

The trend of early initiation of unsafe sexual behaviors among adolescents led them to experience adverse health-related outcomes, including genital pain, genital ulcers/bruises, fungus, teenage pregnancies, STIs, and UTIs. To engage in safe sexual behaviors, adolescents must be given comprehensive and age-appropriate sexual and reproductive health information, education, and directed to age-appropriate healthcare services in health facilities close to but also preferred by them. Nevertheless, a system-wide school and even community-based interventions on matters around sexual and reproductive health to adolescents need to be bolded in national policies, education & health strategic plans, and guidelines for them to reach adulthood while they are healthy and strong enough to produce and contribute to the socioeconomic development of the nation.

### Strength of the Study

In response to SDG3, this study focused on the family planning domain in health, which is a major public concern in the fight against not only STIs/HIV but also teenage pregnancies among young people. This study's findings demonstrated a causal relationship between the variables under study.

### Implications for Practices and Future Research

The study's findings can be used by policymakers, educational institutions, Tanzanian health facility administrative bodies, and people all over the world to develop new approaches for involving and empowering in-school adolescents and caregivers with comprehensive SRH knowledge, attitude, and soft skills for delaying sexual intercourse in their lives. If the findings are published in this prestigious, scientific, open-access journal, they will provide a useful database for in-school large-scale interventions or future research.

### Limitations of the Study

Because the study was limited to a small area, the results may not be generalizable to adolescents from other parts of Tanzania, including those living outside of the coastal region of Morogoro, or from other parts of the nation. Furthermore, the study did not employ a triangulation strategy to gather data, and as a result, the rigor of dependability, transferability, and/or confirmability may not have been adequately addressed, although the results of this study should still be relevant. The ability to rate oneself is challenged because it may lead to under- or over-rating oneself, or describing the habits or data of the female students who participated in the study. Nevertheless, for the students who were nonresidents, the author did not ask them where they slept, whether they shared a room with their parents or slept in separate rooms, and thus, caution may be required when interpreting the study's findings.
